# Illness Experiences of Brazilian People Who Were Hospitalized Due to COVID-19 and Faced Long COVID Repercussions in Their Daily Life: A Constructivist Grounded Theory Study

**DOI:** 10.3390/bs14010014

**Published:** 2023-12-23

**Authors:** Francielle Renata Danielli Martins Marques, Carlos Laranjeira, Lígia Carreira, Adriana Martins Gallo, Wanessa Cristina Baccon, Marcelle Paiano, Vanessa Denardi Antoniassi Baldissera, Maria Aparecida Salci

**Affiliations:** 1 Departamento de Pós-Graduação em Enfermagem, Universidade Estadual de Maringá, Av. Colombo, 5790—Campus Universitário, Maringá 87020-900, PR, Brazil; franrenata.martins@gmail.com (F.R.D.M.M.); ligiacarreira.uem@gmail.com (L.C.); adriana.gallo@ifpr.edu.br (A.M.G.); wanessabaccon@hotmail.com (W.C.B.); mpaiano@uem.br (M.P.); vdabaldissera2@uem.br (V.D.A.B.); masalci@uem.br (M.A.S.); 2 School of Health Sciences, Polytechnic University of Leiria, Campus 2, Morro do Lena, Alto do Vieiro, Apartado 4137, 2411-901 Leiria, Portugal; 3 Centre for Innovative Care and Health Technology (ciTechCare), Polytechnic University of Leiria, Rua de Santo André-66-68, Campus 5, 2410-541 Leiria, Portugal; 4 Comprehensive Health Research Centre (CHRC), University of Évora, 7000-801 Évora, Portugal

**Keywords:** COVID-19, long COVID, adults, older people, qualitative study, Brazil

## Abstract

Long COVID is a multisystem condition that has multiple consequences for the physical, mental, and social health of COVID-19 survivors. The impact of the long COVID condition remains unclear, particularly among middle-aged and older adults, who are at greater risk than younger people of persisting symptoms associated with COVID-19. Therefore, we aimed to understand the experiences of middle-aged and older people who had been hospitalized for COVID-19 and the repercussions of long-term COVID symptoms in their daily lives. A qualitative study was carried out, adopting the framework of the constructivist grounded theory (CGT) proposed by Kathy Charmaz. Fifty-six middle-aged and older adult participants from the southern region of Brazil were recruited. Data were gathered from semi-structured telephone interviews. Concomitantly a comparative analysis was performed to identify categories and codes using the MaxQDA^®^ software (version 2022). Three subcategories were identified: (1) experiencing COVID-19 in the acute phase; (2) oscillating between ‘good days’ and ‘bad days’ in long COVID; and (3) (re)constructing identity. These concepts interact with each other and converge upon the central category of this study: recasting oneself to the uniqueness of the illness experience of long COVID. Our findings provided insights related to the disruption in the lives of long COVID-19 sufferers who still live with persistent symptoms of the disease, including physical, social, family, emotional and spiritual repercussions. Likewise, this study may aid in developing friendly and welcoming social environments, lowering stigma and prejudice towards patients with long COVID, and fostering prompt and suitable policy support and mental health care for these individuals.

## 1. Introduction

The SARS-CoV-2 coronavirus, the agent responsible for the disease COVID-19, was declared a pandemic in March 2020, resulting in a catastrophic event that led to almost 7 million deaths in three years [1]. The United States had the largest number of SARS-CoV-2 infections and COVID-19-related fatalities, followed by India and Brazil [2]. Evidence suggests that male populations from lower socioeconomic backgrounds, and with advanced age, had higher mortality rates from COVID-19 [3,4].

SARS-CoV-2 coronavirus triggers a spectrum of disease severity, from asymptomatic to acute respiratory failure and death. Different clinical patterns were diagnosed, such as mild, moderate, and severe to critical types [5]. Moderate to severe disease cases were often hospitalized and required oxygen support [2]. Although COVID-19 has affected all age groups, older people are more susceptible to the disease because they have more comorbidities and severe inflammatory responses when compared to younger groups [6,7]. A recent systematic review suggests that a more advanced age, the presence of neurocognitive disorders, and impairment or dependence on daily activities are risk factors for mortality from COVID-19 [8].

Three years after the beginning of the pandemic, COVID-19 still represents a global health challenge that needs further clarification regarding its repercussions. An estimated one in five survivors of COVID-19 experience at least one persistent symptom [9]. Prolonged symptoms of COVID-19 occur more commonly in older people [8], and severe infection requires hospitalization [10]. Persistent symptoms (longer than 12 weeks)—known as long COVID—have become a medical and social issue [11,12,13]. Among the hypotheses regarding the etiopathogenesis of long COVID is the possible persistence of the virus in the host; however, the cause is still unknown [14].

According to a recent umbrella review, older age, being female, comorbidities, severity of acute disease, and being overweight or obese may all be related to long COVID-19 [15]. The following signs and symptoms seem to be more frequent, according to three meta-analyses [16,17,18]: tiredness, dyspnea, coughing, sleep problems, anxiety, and depression. Reportedly, fatigue and attention deficit can persist for longer than 12 weeks after COVID-19 diagnosis [16]. Studies also indicate that symptoms of long COVID in older people often include mood disorders, fatigue, anxiety/depression [19] and impairment of quality of life [20]. Other less frequent symptoms may arise, such as myalgia and loss of smell and/or taste [21,22]. A study of older adults with severe COVID-19 requiring hospitalization identified a greater likelihood of having prolonged disease symptoms, especially in individuals who required oxygen therapy [23].

Considering the impact of the pandemic at a global level, understanding the extent of changes and their repercussions is necessary, particularly among those with chronic COVID-19 [24]. A recent meta-synthesis suggests a high burden of biopsychosocial challenges among people with long COVID [25]. The experience of a chronic disease is a complex and constantly changing process, which involves the transition from a previous state to a new condition that requires adaptation by the individual [26]. Research on the lived experience with long COVID highlights its implications for biopsychosocial domains [27] and how an unknown illness can change an individual’s life perspectives. Concurrently, people with long COVID are still stigmatized due to the disease, and most do not feel prepared to take control of their care [28]. According to Mishel’s theory [29,30], managing uncertainty is essential to adaptation during illness, because the persistence of COVID symptoms causes a sense of lack of control over the disease. Following diagnosis, patients are unsure about whether there is a cure, a successful treatment, and what will happen to them and their families. The ambiguity over the illness’ trajectory causes continual stress and anxiety, fear, and despair [31].

Despite the growing evidence on long COVID, there are few qualitative research efforts portraying the lived experience of long COVID sufferers, particularly those who had moderate to severe conditions of acute illness [12,27,32,33,34]. Likewise, there are no known qualitative studies of long COVID in the Brazilian context, which is important based on the assumption that the person’s cultural context affects the disease experience [35]. The interplay between health and culture is latent in how biological, behavioral, and psychosocial factors impact health differences within and among different cultural contexts.

Because long COVID has become a global health burden, we don’t yet fully understand its biopsychosocial impacts, wherein the patient’s role needs to be highlighted. The present study aimed to develop a theoretical model that understands the experiences of middle-aged and older people who had been hospitalized for COVID-19 and the repercussions of long-term COVID symptoms in their daily lives. The research questions were as follows: ‘What are the experiences of middle-aged and older people who have been hospitalized for COVID-19?’; ‘How do they live and manage long-term effects of long COVID in their daily lives?’.

We hope to provide evidence on a little-known reality, informing customized interventions aimed at the needs of survivors and contributing to the desideratum “no one left behind” [36], as underlined by the UN 2030 agenda for sustainable development.

## 2. Materials and Methods

### 2.1. Study Design

This study is part of a larger project designed to evaluate the predictors, sequelae, repercussions, and consequences of COVID-19 in adults and older people who developed moderate to severe forms of the disease after hospital discharge [37].

For this research, a qualitative approach was used, adopting the constructivist grounded theory (CGT) approach [38]. Methodologically, CGT is interpretative and takes a subjective epistemological position to understand the meanings, actions, and processes that people construct and reconstruct, allowing researchers to go beyond a description and construct new concepts that explain that reality [39].

Grounded theory methods help construct a substantive theory that provides a more comprehensive and clear understanding of the core category, subcategories, and connections within a certain subject [38]. For this purpose, individuals who have personally experienced long COVID actively participated in each stage of the research process to improve the quality and relevance of the study.

This study was conducted and reported following the Consolidated Criteria for Reporting Qualitative Research (COREQ) checklist [40].

### 2.2. Setting, Participants and Recruitment

The study was conducted in a state in the southern region of Brazil. Although this region has more hospital resources compared to other regions of the country, its COVID-19 mortality rates were higher than other regions with poorer medical services [41], thus justifying the recruitment of people who developed long COVID in this context.

Participants were selected according to an intentional sampling technique using the telephone records of individuals who had developed acute COVID-19 and who were registered in the databases “Notifica COVID Paraná” and “Information System for Epidemiological Surveillance of Influenza (SIVEP-Influenza)”.

Study participants included middle-aged (aged 45 to 59) and older (aged 60 or older) people, according to the categorization proposed by the WHO [42], considering that the risk of developing long COVID increases with age [9,43].

All participants were recruited regardless of sex, race, occupation, and acute COVID-19 severity to obtain the maximum variation in the sample. Inclusion criteria for eligible participants were (a) age ≥ 45 years; (b) having had COVID-19 classified as moderate or severe in the acute phase; (c) having one or more symptoms of long COVID for up to 9 months after infection with SARS-CoV-2 (time of data collection); (d) being able to communicate and understand Portuguese; and (e) having responded to telephone contact (up to three attempts). Pregnant and puerperal women were excluded.

The number of participants reached theoretical saturation when no new evidence was found in the face of conceptual exploration [39]. Theoretical saturation contributes significantly to study quality by strengthening researchers’ analysis and providing them with material to make explicit claims [39].

### 2.3. Data Collection

Data collection lasted four months and was carried out between May and August 2022. Potential participants with long COVID-19 were contacted via telephone. During the initial contact, the objectives of the study and the importance of their participation were explained. After acceptance, the Informed Consent Form was sent by the means requested by the participant (letter or email) and the interview was scheduled on a day and time defined by the participant. Participants had up to 48 h to return the signed consent on paper via mail before the interviews took place. No time limit was established for the decision to participate.

The interviews were conducted using a semi-structured guide, built based on the available literature [32,44]. The guide was later validated by three PhD researchers with expertise in the subject. The open questions dealt with the experience of hospitalization due to COVID-19, long COVID’s trajectory, the facilitating and inhibiting factors experienced with long COVID, and the coping strategies used. Participants were encouraged to delve into their answers using conversational prompts (e.g., “How did you feel?”; “Tell me a little more about this”; “Can you give examples?”).

After preparing the script, a group of researchers, composed of seven nurses (master’s or doctoral students in nursing), was instructed and trained in qualitative interviewing (a course with 40 training hours). Training in interview facilitation skills included establishing trust and rapport; active listening; adopting a nonjudgmental approach; using open-ended and probing questions; memoing and reflection; and minimizing interviewer bias [45]. None of the researchers were involved in the actual care of the subjects. Field notes on the researchers’ perceptions were carried out during and after the interviews [38]. The interviews lasted between 30 min and 90 min (average duration of 45 min) and were recorded in audio and transcribed in full. There were no repeat or follow-up interviews. In compliance with the assumptions of the framework [38], the topic guide was adapted during data collection as new themes emerged.

### 2.4. Analysis

Data collection and analysis were carried out simultaneously, using a constant comparative approach throughout this process [38]. This helps researchers to constantly focus on developing and deepening concepts from the data [39].

First, the initial coding was performed. Data were inductively coded, line by line, with emphasis on important concepts and phrases. Next, focused coding was carried out to visualize the connections between categories. The last stage of coding was integration, which develops increasingly from the beginning of the analytical process until all categories are saturated, sustaining the phenomenon. The categories were organized around a central concept called the core category. This explanatory model derives from the researcher’s ability to combine theoretical sensitivity with the inductive–deductive process to generate the theory that explains the phenomenon under study [46].

The MaxQDA^®^ software (version 2022) [47] was used to file and manage qualitative data during the coding stages. To ensure that extracts were translated accurately, they were first translated into English and then back into Portuguese. Sample quotes were presented according to interview order (e.g., P1…, P56) and age.

### 2.5. Study Rigor

Charmaz’s criteria of credibility, originality, resonance, and usefulness were used to ensure the study’s rigor [38]. The use of CGT ensures transparency in what researchers learned, showing how the research was conducted in a complete and systematic way [39].

Credibility was strengthened by training researchers to collect data and by peer reviewing the analysed data to obtain a consensus and mitigate any potential bias. Credibility also involves reflecting on the researcher’s views and actions throughout the entire data collection and analysis process., which is one of the strategies that ensures that the study’s findings are the product of the research’s focus and not a mere subjective interpretation by the researcher. Since this research was anchored in the constructivist approach, the researcher’s reflexivity played a fundamental role in how the data were discovered. The main researcher is a Brazilian white female (F.R.D.M.M.), with a background in nursing and a research focus on chronic illnesses and the organization of health systems; currently, she holds the position of faculty member. The other team members included nurses working with clinical populations with chronic conditions (W.C.B.; A.G.) and researchers with prior experience of conducting qualitative health research (C.L.; L.C.; M.P.; V.D.A.B.; M.A.S.).

Originality and resonance of the findings were both obtained through continued interviews (until data saturation) and the use of the constant comparative method as a way of validating emerging information. The constant comparison between the coded concepts generated the central category, identified by an iterative process. The MaxQDA^®^ software allows the organization of the data and creates a detailed script so that a properly trained auditor can assess the entire process. The theory emerged based on the central category, which comprises the main phenomena, allowing comprehension which allowed its construction to be comprehensive. The theory was generated through discussions with the study’s other researchers and using memos and diagrams (usefulness criteria) [39]. Finally, usefulness also includes forming a foundation for practice applications and new lines for further research.

### 2.6. Ethical Considerations

The study was performed according to the principles of the Helsinki Declaration. The protocol was approved by the State University of Maringá—UEM’s Research Ethics Committee (Opinion No. 4.518.26). Before each interview, written informed consent (including permission to record audio) was granted. Participants were informed they could opt out of the study at any time. They were not compensated for their participation.

## 3. Results

### 3.1. Participants’ Characteristics

The sample included 56 people, aged between 45 and 88. Participants were predominately male (middle-aged adults = 64.3%; older adults = 71.4%), white (middle-aged adults = 71.4%; older adults = 67.9%), living with a partner (middle-aged adults = 78.6%; older adults = 85.7%), and Catholic (middle-aged adults = 60.7%; older adults = 67.9%).

Regarding their clinical characteristics, participants were mostly middle-aged adults (64.3%) hospitalized for severe acute COVID (bilateral pneumonia and respiratory failure needing mechanical ventilation). Among the older sufferers, there was no difference between moderate (bilateral pneumonia needing high-flow oxygen support) and severe manifestations (33.3%). Most middle-aged adults were hospitalized for up to seven days (32.2%); older people, from 15 to 30 days (39.3%). Long COVID symptoms lasted between three and six months (middle-aged adults = 57.1%; older adults = 75.0%). The most prevalent long COVID symptoms reported in both groups were shortness of breath/tiredness followed by muscle aches. Table 1 presents a description of the study participants’ characteristics.

### 3.2. Findings from Interviews

**Figure 1:**
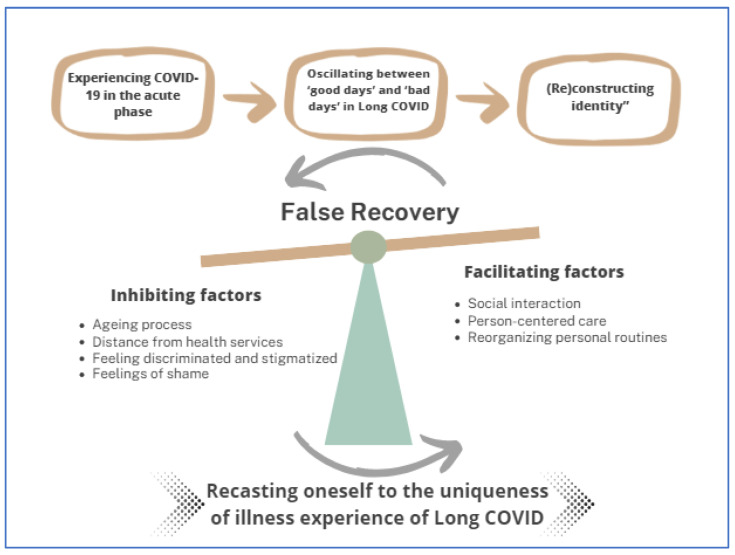
Overview theory: Illness experience journey from COVID-19 hospitalization to the long-term effects of long COVID on the daily lives of their survivors.

Data analysis identified three subcategories: “Experiencing COVID-19 in the acute phase”; “Oscillating between ‘good days’ and ‘bad days’ in Long COVID”; and “(Re)constructing identity”. These subcategories interact with each other and converge on the central category of this study: “Recasting oneself to the uniqueness of illness experience of Long COVID”. An overview of the categories and subcategories is shown in Figure 1.

The experience of long COVID is highly unpredictable in people living with chronic health problems and can persist for weeks to months after COVID-19 infection, leading to a variety of biopsychosocial difficulties that affect relationships and daily life. Hence, many individuals realize they are different from before they had COVID-19 and consequently deal with existential loss. Overall, participants grieved for their old selves and integrated their new selves in a sequence of adaptation and integration.

#### 3.2.1. Experiencing COVID-19 in the Acute Phase

The experience of being hospitalized due to COVID-19 was frightening for all participants. For some, hospitalization accentuated their fear of death, having experienced the loss of friends due to the disease; for others, the feeling of anguish was intensified during hospitalization by a kind of “fear marketing” provoked by the media.

*Wow, I was really scared when the doctor told me it was COVID. Because I had already lost a friend to the disease; so for me, it had another significance.*

*(P51; 58 years old)*

*We saw a lot of sad things on TV. People who are hospitalized and never leave there alive.*

*(P31; 63 years old)*

*I felt a lot of fear, a lot of sadness, a lot of fear of dying. Because there was news of deaths every day. I couldn’t leave the house and I can’t turn on the TV because that was all we talked about. So, when I was admitted, I knew it was serious.*

*(P12; 69 years old)*

Regardless of whether hospitalization took place in medical wards or the Intensive Care Unit (ICU), the life-threatening experience was constant and generated existential suffering. The narratives report a fear of imminent death, the observation of loss in other people hospitalized in nearby beds, and a feeling of impotence before the evolution of the disease; all described as stressful events during hospitalization.

*It was terrible because I had had surgery before, and had gone through procedures, but it was scheduled. […] I was very afraid of dying because when I was hospitalized, I was already short of breath. Then I got worse and had to go to the ICU. So, I was afraid I wouldn’t come back from there.*

*(P22; 45 years old)*

*It was very traumatic to see so many people dying by my side and there was no follow-up to change it, there was no psychologist, psychiatrist, nothing! You never get that out of your mind [...] looking to the side and seeing the person there, agonizing, dying beside you. That we will never forget. In the morning, one would die; in the afternoon, another would die, and so it went.*

*(P36; 66 years old)*

*There was a lot of suffering, how I suffered! And I remember being hospitalized, but I was getting worse, and I couldn’t do anything, I couldn’t do anything… And when the doctor said they were going to transfer me to the ICU, I thought it was the last time.*

*(P37; 65 years old)*

Although necessary, isolation during the confinement period resulted in restrictions on hospital visits, hindering family support during hospitalization. In addition, the use of personal protective equipment, particularly masks, limited interacting by using nonverbal language, increasing the feeling of isolation in the participants.

*I was hospitalized for a long time, alone, without visitors and we missed having someone to talk to.*

*(P34; 61 years old)*

*Even the family couldn’t come to see me properly. We know this isolation was necessary, but time did not pass, and [I spent] a lot of time alone.*

*(P56; 37 years old)*

*When someone entered the room, they were all dressed up. We didn’t even see the faces of those who came to examine us.*

*(P55; 71 years old)*

Information and digital communication technologies were used to bring family members closer together, keep their family bonds ‘alive’ and minimize isolation, homesickness, and the stress caused by hospitalization. This reality was particularly relevant when, due to the ageing condition, the spouses could not carry out a face-to-face visit due to the increased risk of infection. The findings reinforce the idea that COVID-19 was an illness for the whole family, given that all family members suffered its consequences.

*I spent 45 days watching [my daughter] only on video. But it was just as well because she could come to see me and get the disease. But it was very difficult. It’s a good thing that nowadays there is this technology (video call), so I could quench my nostalgia a little.*

*(P27; 46 years old)*

*Those who came were my children because my wife is elderly too, so she was also at risk. So, there was no way for her to come. Then, after I left the ICU and went to the room, they called me so I could say that everything was fine and that I was recovering because she also suffered a lot. Everyone suffered.*

*(P53; 69 years old)*

After hospital discharge, participants still had a plethora of persistent symptoms, many of which were related to breathing impairments and the need for bed rest. They reported feelings of a deregulated body due to the functional limits imposed by the disease. During this period, family support was important for rehabilitation, particularly in cases where, due to the lack of adapted homes, participants were forced to resort to other alternatives.

*I needed a lot of help, because when I was discharged from the hospital, I still couldn’t walk, I was tired. So, I had to come to my sister’s house to do physical therapy and be able to walk again. Where I live is an apartment, there are stairs, and I was unable to recover there. Thus, my sister had a space in her house so that I could do all the recovery.*

*(P15; 33 years old)*

*As soon as I was discharged, I had a little difficulty recovering my balance, I was shaking a lot. The recovery was a difficult time, for about five months or so. During this period, my family helped me, they stayed at home to help me if I needed it. Sometimes I stayed in bed and my children took turns caring for me. I wouldn’t have made it without them.*

*(P23; 81 years old)*

#### 3.2.2. Oscillating between ‘Good Days’ and ‘Bad Days’ in Long COVID

The insidious and fluctuating nature of long COVID symptoms was described by the participants as a false recovery, wherein there were “bad days” when they were forced to live with exacerbated symptoms, bringing repercussions on their daily activities, and “good days” when symptoms were mild or non-existent, causing them to feel recovered.

*Some days we are fine, some days we are not. Some days I wake up early, clean the house, make lunch, wash clothes, wow… I do everything. There are other days when I can barely walk because of breathlessness.*

*(P16; 49 years old)*

*The days I am more attacked *[by forgetfulness]*, if I go there to save something and turn my back, I no longer know where I put it and I keep looking. There are days when it even gets in the way of driving. Sometimes, in the truck itself, I’m on the road and I wonder where I am.*

*(P10; 60 years old)*

Participants described both inhibiting and facilitating key influencing factors in living with long COVID recovery. 

##### Inhibiting Factors

Older people commonly use the expression “normal for their age” to justify the persistence of long COVID symptoms, which results in the person feeling less responsible for taking care of their health. This use reflects a double transition that elderly participants were experiencing: ageing and long COVID.

*I got a lot worse after COVID; it’s not like it used to be. There’s always a weakness, a bit of forgetfulness, but I think it’s normal, it’s really [that I’m] getting old.*

*(P26; 72 years old)*

*I got more nervous and more stressed, but I think it’s age too.*

*(P11; 77 years old)*

*I don’t know if it’s because of my age, because I’m getting old, but my eyesight is also altered, and all that came after COVID. I felt that my eyesight was weakening, and I use it a lot in my work because I travel by car, so I strain my eyes a lot.*

*(P7; 68 years old)*

The distance to health services was a barrier that hindered the recovery of some participants. The fact that they identified the symptoms of long COVID-19 as normal in the ageing process demonstrated little adaptive behavior by the participants, namely in the search for differentiated health care.

*I didn’t go [to the doctor] anymore to find out if it was COVID, but this forgetfulness, this weakness is something of old people, that’s why I didn’t go.*

*(P29; 77 years old)*

*I didn’t go to the healthcare unit because we see that even *[professionals]* don’t really know what to do with us. They are also trying to understand this disease.*

*(P35; 79 years old)*

*My wife says that I got slower, that my mind got slower, but I haven’t been to the doctor yet to find out if this is due to COVID, I think it’s the dizziness of older people. Forgetfulness and these things, I think it’s due to advanced age.*

*(P19; 65 years old)*

Another negative factor experienced by the participants is the feeling of discrimination resulting from the disease. Moments were reported when they felt “reduced” in their personhood and stigmatized due to their condition of being a “Long COVID sufferer”, which limited social interactions.

*I’m more antisocial now, I want to be more alone, and isolated. I used to have a family, and now I don’t, I live alone. I know why I felt like that, because when you’re in the hospital everyone is on top of you, it seems like affection for you, but in fact it’s not affection, it’s like pity.*

*(P52; 49 years old)*

*I even got mad at my daughter because she told the neighbours that I was re-hospitalized due to Long COVID symptoms. I didn’t want her to tell anyone because everyone here was looking down on us. And then, the neighbours would greet us on the street, and they would talk to us at the gate, but they wouldn’t come into the house anymore.*

*(P38; 81 years old)*

On the other hand, the recurrent episodes of forgetfulness and frustration from not being able to justify the persistent COVID symptoms generated feelings of shame when interacting with others, aggravating the level of social isolation. Some participants mentioned the following issues:

*It took me a while to want to leave the house again because I had a lot of complications. And this forgetfulness; we feel ashamed when talking to others.*

*(P46; 61 years old)*

*We see people who have had COVID just like we had, but who are fine. And it seems that only you are not. So, people keep asking ‘why we don’t get better’ as if it were my fault (...) That’s why I ended up becoming less sociable because that’s frustrating.*

*(P50; 54 years old)*

##### Facilitating Factors

For some participants, social interaction promoted “good days” during the experience of persistent symptoms and helped them positively face the changes that occurred in their lives.

*I have many friends, so as soon as I was discharged, my neighbours would come to my house to see me, talk to me, and see if I needed anything. That was good because we feel loved, knowing that so many people are rooting for us; it helps.*

*(P1; 79 years old)*

*Even though I still can’t get out of the house much, when I can, I always go to Church group meetings. It’s good for us to go out, talk a little, and laugh, it makes everything happier.*

*(P3; 66 years old)*

The search for health care was also indicated as a facilitating factor for some participants, as they experienced person-centered care based on their needs, which allowed them to facilitate recovery.

*I was so forgetful that I went into depression. I cried every day; I felt like I was losing my memory. And that didn’t go away and it distressed me. My family took me to the psychiatrist almost by force (laughs). Because we never think we need it. So now [with the treatment], it has improved a lot. I already feel recovered.*

*(P13; 69 years old)*

*Look, I was treated very well at the hospital and that also motivated me to want to get better. I saw that *[the healthcare professionals]* were there doing everything to save me, every time someone came to examine me, ask how I was doing, and talk about my exams. So that motivated me to improve. Even on the worst days of the symptoms, I struggled because I saw that the professionals were helping me.*

*(P42; 58 years old)*

Looking for health recovery during the chronic phase of COVID required adaptive strategies by the participants, signaling their desire to regain control of their lives and ability to move forward. One of these strategies was the search for daily normality. Establishing a routine for domestic and work tasks was the result of the search to reorganize their activities, an attempt to overcome the barriers imposed by the disease, as stated below:

*I’m self-employed, so I’ve had to adjust my work schedule. I can no longer work long hours straight because I feel very tired. Therefore, I needed to adjust this. I take a lot of breaks to get work done again.*

*(P49; 50 years old)*

*I changed my routine at home. So even to take care of the house, I had to change. I used to be able to do all the house cleaning in a single day, but now I can’t anymore. So, I moved on to splitting my cleaning chores.*

*(P43; 46 years old)*

#### 3.2.3. (Re)constructing Identity

Intrapersonal resources—such as gratitude for life in the present moment and hope for the future—were used as post-traumatic growth strategies. These changes are perceived as positive by the individual after experiencing a negative event such as “living with COVID-19”. Resilience also emerged as the ability of individuals to create or maintain health and well-being even in the face of adversity.

*I recovered quickly because I believed that I would get better because my illness was not that serious. I said that to myself, I thought about getting better, I only thought about good things.*

*(P33; 70 years old)*

*I saw [survival] as a chance at life. Even if I still have some sequelae and a little shortness of breath, I know the worst is over. It’s a hope I have.*

*(P39; 54 years old)*

*I keep thinking about how much I still have to improve, but when I think about how many people died, I stop thinking about these bad things for a time. So, I guess I should be grateful for this chance I had and of course hope for the future.*

*(P45; 54 years old)*

*I always believed I would get better; I think good things attract good things and the universe works in your favour.*

*(P4; 50 years old)*

Participants indicated that surviving COVID-19 allowed them to reassess their priorities. There was time to rethink their lives, attitudes, and projects for the future, particularly in areas such as work–life balance, *Carpe diem*, and self-care.

*I was a person who worked too hard and never had time for anything, not even to take care of my health. I still have a lot of sequelae, so I worry more about my health now.*

*(P14; 47 years old)*

*These moments of recovery right after discharge were important because we see that we do so many things on a daily basis that we don’t have time to think about ourselves, think about life. I was thinking about the life I had and the life I wanted to have from now on.*

*(P40; 58 years old)*

*I have now learned not to leave anything for tomorrow. Because today I’m fine, I’m here alive and recovering, but tomorrow I might not be. Just like when I got COVID; I was fine one week and hospitalized the next. So now, if I feel like going out, going to a park, or getting some fresh air, I will. I won’t do it on Sunday if I can do it in the middle of the week.*

*(P2; 66 years old)*

In the same sense, other participants signaled changes in their perspectives on life, on what they now consider to be important, describing the strengthening of family and professional ties as the highest priority.

*I had many consequences, I had to start from scratch. But on the other hand, it was good because it strengthened my relationship with people a lot. I could see who was on my side, my sister, and my family, and that helped me a lot and strengthened me a lot. The lesson I see in this is that “Look, you had a second chance, let’s do it differently?” Hence, I take it as a lesson.*

*(P18; 51 years old)*

*The whole family got closer. If there’s one thing we can learn from all this, it’s family. Because even after I was discharged, it took me a while to recover properly, and I still haven’t returned 100%. But everyone helped. Even people farther away from the family, whom we hardly spoke to before, now call me. So, I think that it was a good thing that I stayed.*

*(P20; 72 years old)*

*I had to change jobs due to the consequences of COVID. And it was nice to see that this mobilization came from them. I didn’t have to ask or say that I couldn’t do it. They arranged everything at work for when I arrived.*

*(P9; 62 years old)*

Some participants demonstrated that they had become more compassionate people. Having experienced a life-threatening illness made them more sensitive to the suffering of others. In this regard, P24 referred to having started to “look at the world with different eyes”, translating self-knowledge and mutuality into the experience of human suffering.

*We start to see the world with different eyes, that’s the truth. What we never had time to do, now we want to do. Because we see that we are nothing without health.*

*(P24; 51 years old)*

*I was very emotional; I wasn’t like that before and I was afterwards. Now I cry easily. Especially if someone comes to tell me that they also almost died; I keep remembering everything I went through too, thinking that I managed to survive.*

*(P30; 59 years old)*

However, one participant alluded to the stereotyped way in which men and masculinity are represented in the Brazilian context, centered on the notions of autonomy, strength, and power; in contrast to the vision of femininity, based on softness, the expression of emotions, and dependence. In this sense, P44 referred to having resignified his mindset from a logic of suppression of feelings to an appreciation of dialogue and affection.

*I came from an upbringing where men don’t cry, so I had never cried in front of my family. Today you can’t show a report on TV, about disasters, about people who died, because I cry too. I seem to feel more.*

*(P44; 69 years old)*

Based on the disease’s trajectory, most participants report having strengthened their faith. In the reports, one can perceive that belief in the divine had a protective role in overcoming the obstacles associated with the disease, facilitating the connection with oneself and with others in the search for reconstructing identity.

*What I learned looking back is that the world wouldn’t stop if I died. So now I’ve learned to prioritize myself.*

*(P17; 45 years old)*

*I was practically dead! I’ve always been a person of great faith, so I think that helped me stay alive. And now too, in my new life.*

*(P5; 69 years old)*

*Even though I survived hospitalization, I felt a lot of sadness, a lot of anguish, because I wanted to recover quickly and do the things I used to do before, and I couldn’t. So, I got attached to God, and I asked him to heal me so that I could be me again. What I could do was pray, I couldn’t do anything else.*

*(P25; 78 years old)*

Religious practices acted as mediators of the positive confrontation with long COVID. Brazil is a predominantly Catholic country, and religious practices—such as praying and going to mass—are considered catalysts for dealing with suffering.

*I was baptized (…) I talked a lot with God; I asked him to let me live and I still ask him to help me recover. Because I never stopped believing in Him.*

*(P32; 75 years old)*

*I’ve been praying a lot, asking God a lot for all this to pass soon so that I can win this battle for good. I’ve already improved a lot, but I still have to improve more. Thus, praying comforts me, and gives me hope to recover.*

*(P28; 53 years old)*

*Faith makes us accept what we cannot change.*

*(P48; 81 years old)*

## 4. Discussion

To the best of our knowledge, this is one of the first attempts to gather qualitative accounts of the illness experiences of Brazilian people who were hospitalized due to COVID-19 and faced long COVID repercussions in their daily life, with the aim of developing theory from the data. Our findings revealed difficult experiences associated with hospitalization due to COVID-19, as well as the challenges that persistent COVID symptoms represent in the lives of individuals pursuing daily life activities.

The experiences expressed by the participants regarding the acute phase of the COVID-19 disease trajectory were similar to those reported in other studies, which describe several negative emotions such as stress, fear of death, and concerns about the course of the disease [27,48,49]. Likewise, Sahoo et al. [50] found that many COVID-19 survivors had experienced mental suffering because of isolation, a fear of dying, and related stigma. One reason for emotional intensification was the difficulty of nonverbal communication with the healthcare team during hospitalization, as their use of personal protective equipment limited touching, facial expressions, and smiling [51]. Likewise, evidence suggests that stress and fear of COVID-19 were fueled by the media, given the “disinfodemic” instilled by either the large amounts of fake news or by the social panic generated by the daily dissemination of statistics on infections and mortality [49,52,53].

Since patients felt that isolation was required by the restrictions due to the pandemic, loneliness may not have been reported in our study. Isolation was not seen as a bad thing because of the availability of mobile phones and trust in professionals [54]. Maintaining dialogue with family members, even over the phone, made it possible to alleviate the stress caused by hospitalization. Our findings and those of other studies have also indicated that the disease could be seen as an opportunity to strengthen ties and improve family relationships [49]. There was a need to establish new ways to connect patients and family members who were physically absent from the bedside [55]. Visiting restrictions in the hospital environment were necessary to contain the spread of the virus, but social distancing need not imply social disconnection [56]. Although older people may have had some difficulty dealing with videoconferencing applications, the use of technologies proved to be satisfactory for maintaining well-being and connection with family and friends [57].

Our findings also reinforce the perspective of COVID-19 as a familial disease. Although, in some cases, only one member of the family was affected by the disease, the whole family suffered its consequences. The hospital admission of a loved one causes changes in the structure and functioning of the family, generating feelings of shock, uncertainty, denial, anger, despair, hope, guilt, anxiety, and fear of the death of the family member, an experience almost always described as traumatic [58]. The changes in family life caused by COVID-19 affected employment, generated financial instability, harmed the mental health of family members, and required efforts to balance external stressors in order to develop coping strategies to deal with the difficulties inherent in the pandemic and, eventually, achieve a level of family well-being [59].

Surviving acute COVID-19 affected the experience of living with long COVID’s intermittent symptoms, generating a feeling of false recovery. Although intermittent post-COVID symptoms are present in all clinical forms of COVID-19, the frequency is higher in those with moderate and severe forms [60], which justifies the representativeness of this subcategory in our study. Long COVID as a chronic condition leads to uncertainty about the future, causing the subject to mobilize resources in search of self-recovery, in an attempt to escape the sick role due to the stigma associated with long COVID and reinforce their identity [27,61]. Long COVID is said to be the first illness that patients created collectively [13]. Patients who were first diagnosed with this illness had inexplicable symptoms and often felt untrusted and neglected by others. In this sense, Poyraz et al. [62] highlight that inadequate social support is associated with the occurrence and severity of PTSD symptoms, with protracted symptoms, wherein the public and self-stigma attached to long COVID are important predictors of PTSD.

As illustrated in the current study, participants often associate persistent COVID symptoms with the physiological consequences of ageing. Evidence suggests that symptoms of long COVID may be difficult to distinguish from changes associated with ageing, particularly in older adults with high rates of comorbidities and polypharmacy [43]. Since older individuals have higher rates of long COVID [22], the atypical presentation of clinical manifestations in elderly people may influence the pattern of symptoms at disease onset. Overall, these findings confirm that the identification of long-term medical conditions, such as long COVID, may be more problematic in older people and individuals with high clinical complexity [63].

Since the absence of effective treatments fuels the frustration of long COVID sufferers, peer support programs and other relational approaches can potentially improve scores on medically determined outcome measures and foster psychosocial and advocacy benefits [64]. However, the fragmentation of health systems limits peer support effectiveness because they are still recovering from the disruption caused by the pandemic [28]. Hence, anti-stigma practices related to COVID-19 must be implemented urgently to minimize experiences of discrimination and facilitate their identification by health services to ensure proper management [65].

The chronicity and possibility of relapse imposed by long COVID can also lead to the loss of jobs, income, and social interactions. In turn, this can lead to mental health problems [66]. Regarding this issue, Kohn et al. [67] stress the value of educating employers on the difficulties associated with long COVID.

In the search for recovery from long COVID, intrapersonal resources such as gratitude, hope, and resilience were identified by participants as strategies to preserve health and well-being, even in the face of adversity. Given these benefits, understanding how hope and resilience develop and thrive is significant, for they will aid in the development of hope-based interventions [68]. The illness experience contributed to a reassessment of people’s lives, priorities, and well-being, which contributed to the experience of personal growth [65,69].

When someone’s life journey is challenged by a serious illness, people need to try to find a way to keep moving forward [70]. The perception of being/feeling sick and moving on requires the incorporation of new knowledge, the identification of inhibiting and facilitating conditions, and behavioral change in the face of what affects their health status [71].

The work–life balance was also identified by our participants as a need during recovery. The adaptive changes made in the work environment—whether in schedule flexibility or the possibility of working from home—provide a balance between personal and professional life, as well as in the relationship with work [72]. Changes in work patterns during this period influenced employees’ work–family balance, which in turn affected their adjustment and job satisfaction [73].

This study identified a (re)discovery of self-care, which contributes to the participant’s post-traumatic growth. Self-care is not just for personal benefit. There is a cascade effect of well-being in the environment in which it is developed, since the self-care of some individuals can collectively impact and influence others [74]. Therefore, the implementation of interventions aimed at promoting resilience and self-care is suggested to mitigate the impact of the pandemic on patients and families [75].

Existential thoughts about life’s meaning and purpose often affect people who experience the long-term effects of COVID [76]. Inner religiosity and spirituality were potential sources of strength and hope in coping with the disease, mitigating the mental suffering of those involved [77,78]. Our findings revealed that the search to restore identity during long COVID was mediated by religious coping for many participants. The strengthening of faith and religious practices was related to greater life satisfaction and well-being. Similar findings were found by Sem et al. [79], who reported that COVID-19 survivors using spiritual/religious resources obtained comfort and reliance on a high power, which may generate existential growth.

### 4.1. Study Limitations

This research used qualitative interviews rather than just quantitative data, producing a deeper level of information and a closer approach to participants’ real-world experiences. However, there are several limitations in this study, including a convenience sample of middle-aged and older sufferers, only recruited through telephone calls. As we did not include those without access to this technology, there may be additional themes that reflect the lived experiences of those populations not included in this study. The telephone is commonly used in Brazil for telemarketing scams and manipulating or tricking users. This may have promoted distrust during the initial approach. Furthermore, interviews by telephone represent a risk of bias due to lost information related to non-verbal communication. However, we emphasize that its use allowed access to a sample of participants from different southern parts of the country. Despite this, data transferability is limited to other Brazilian states with different epidemiological magnitudes of long COVID.

While this study included several sociodemographic variables, it did not consider the social disparities that can affect long COVID. Factors such as comorbidities, cultural diversity, housing conditions, income, and health inequalities are factors that could not be related to our findings, but which we understand can influence personal experiences. In addition, participants classified with mild COVID-19 in the acute phase were also not included, as we understand that these people would be less relevant for assessing the repercussions of long COVID. However, we stress the need for future studies to understand the consequences of long COVID in this subgroup.

Participants were middle-aged and older adults. However, the low representation of those aged over 85 years does not allow the transferability of our findings to this population. Moreover, all our participants had long COVID, but not all had the same duration of symptoms, which may be reflected in their perceptions of the impact of the disease on their living conditions and health.

Lastly, as data collection required the recall of information from events that occurred up to nine months prior to collection, a recall bias may have arisen. It should also be noted that both long COVID and ageing can cause memory lapses, which may have interfered with the quality of our findings.

### 4.2. Implications for Practice

The implications for clinical practice lie in the development of health surveillance to minimize underreporting of long COVID and systematically support self-care and planning for the resumption of daily activities to improve the quality of life of people with long COVID. Supporting people living with long COVID requires specialized, adaptable, flexible, multidisciplinary, and patient-centered care [80]. To tackle this complex condition optimally, the perspectives and experiences of persons with long COVID should continue to be investigated to inform models of care and services developed/adapted for people with long COVID [81].

To reduce instances of medical gaslighting and its detrimental effects on people, healthcare professionals need to be educated on how to deal with ambiguous symptoms and enhance patient involvement. Furthermore, to avoid a crisis of long-term, chronic illness brought on by the early mishandling of the pathology and its possible effects on health systems and economies, comprehensive long COVID recommendations are crucial. Based on our findings, we also suggest the need to expand studies with longitudinal mixed methods, since long COVID is experienced in a persistent and often fluctuating manner.

Living with the persistent symptoms of COVID impacted the lives of middle-aged and elder sufferers, challenging them daily to acquire skills to survive and restore their well-being. Only by grasping the entire construction process of such complex and individualized experiences can we foster more holistic responses and support the existential restoration of identity in long COVID [76]. Thus, effective interventions to support mental health and address patient care needs are required. Finally, the lived experiences of long COVID need to be heard and supported through education, research, and advocacy, which is possibly the most crucial action.

## 5. Conclusions

Grounded theory allowed us to explain the illness experience journey from COVID-19 hospitalization to the long-term effects of long COVID on the daily lives of survivors. We identified three main subcategories: “Experiencing COVID-19 in the acute phase”; “Oscillating between ‘good days’ and ‘bad days’ in Long COVID”; and “(Re)constructing identity”. During this trajectory, long COVID sufferers experienced difficulties in seeking recognition and support from others, but this support, when received, also helped them organize their daily lives and manage uncertainty. In the search for “Recasting oneself to the uniqueness of illness experience of Long COVID”, coping strategies were used to recover well-being and reframe the self. Despite the negative consequences for the life and health of people with long COVID, positive experiences were reported, such as the reorganization of life priorities revealed by the participants. Thus, we hope that these findings will be recognized by health professionals and used in the management of people with long COVID to improve their coping skills. Furthermore, we also expect to inform research seeking to understand the biopsychosocial impacts of long COVID and help patients and their carers access healthcare services and psychological support.

## Figures and Tables

**Table 1:** Sample description (N = 56).

Variables	Middle-Aged Adults (n = 28)	Older Adults (n = 28)
Age (years)		
Mean (SD; range)	52.05 ± 5.15 (45–59)	68.36 ± 7.29 (60–88)
Sex, n (%)		
Male	18 (64.3)	20 (71.4)
Female	10 (35.7)	8 (28.6)
Race, n (%)		
White	20 (71.4)	19 (67.9)
Black/African descent	8 (28.6)	9 (32.1)
Relationship status, n (%)		
Living with partner	22 (78.6)	24 (85.71)
Single	6 (21.4)	4 (14.29)
Occupation, n (%)		
Retired	1 (3.5)	23 (82.1)
Employed	15 (53.6)	5 (17.9)
Unemployed	12 (42.9)	0
Religion affiliation, n (%)		
Catholic	17 (60.7)	19 (67.9)
Evangelic	9 (32.2)	9 (32.1)
Spiritist	2 (7.1)	0
Acute COVID-19 severity, n (%)		
Moderate (bilateral pneumonia)	10 (35.7)	14 (33.3)
Severe (bilateral pneumonia and respiratory failure)	18 (64.3)	14 (33.3)
Length of hospitalization stay (acute phase), n (%)		
up to 7 days	9 (32.2)	6 (21.4)
7 to 15 days	8 (28.5)	7 (25.0)
15 to 30 days	6 (21.4)	11 (39.3)
30 to 60 days	5 (17.9)	4 (14.3)
Long COVID symptoms, n (%)		
1 to 2 symptoms	8 (28.6)	8 (28.6)
3 to 4 symptoms	14 (50.0)	16 (57.1)
5 to 7 symptoms	16 (21.4)	4 (14.3)
Long COVID most reported symptoms, n (%)		
Shortness of breath/Tiredness	10 (35.7)	11 (39.3)
Muscle aches	10 (35.7)	10 (35.7)
Hair Loss	5 (17.9)	6 (21.4)
Sadness/Anxiety	6 (21.4)	6 (21.4)
Memory loss	5 (17.9)	7 (25.0)
Loss of appetite	6 (21.4)	4 (14.3)
Smell change	5 (17.9)	4 (14.3)
Taste change	2 (7.1)	6 (21.4)
Motor change	2 (7.1)	3 (10.7)
Long COVID symptoms duration, n (%)		
3 to 6 months	16 (57.1)	21 (75.0)
6 to 9 months	12 (42.9)	7 (25.0)

## Data Availability

This paper is a part of the doctoral thesis of the first author, and all data generated or analysed during this study are included in this article.

## References

[1] World Health Organization (2023). Coronavirus Disease (COVID-19) Dashboard.

[2] Cascella M., Rajnik M., Aleem A., Dulebohn S., Di Napoli R. (2023). Features, Evaluation, and Treatment of Coronavirus (COVID-19). StatPearls.

[3] Maness S.B., Merrell L., Thompson E.L., Griner S.B., Kline N., Wheldon C. (2021). Social Determinants of Health and Health Disparities: COVID-19 Exposures and Mortality Among African American People in the United States. Public Health Rep..

[4] Shang W., Wang Y., Yuan J., Guo Z., Liu J., Liu M. (2022). Global Excess Mortality during COVID-19 Pandemic: A Systematic Review and Meta-Analysis. Vaccines.

[5] Qi J., He D., Yang D., Wang M., Ma W., Cui H., Ye F., Wang F., Xu J., Li Z. (2021). Severity-associated markers and assessment model for predicting the severity of COVID-19: A retrospective study in Hangzhou, China. BMC Infect. Dis..

[6] Ayoub H.H., Chemaitelly H., Seedat S., Mumtaz G.R., Makhoul M., Abu-Raddad L. (2020). Age could be driving variable SARS-CoV-2 epidemic trajectories worldwide. PLoS ONE.

[7] Shanbehzadeh S., Zanjari N., Yassin M., Yassin Z., Tavahomi M. (2023). Association between long COVID, functional activity, and health-related quality of life in older adults. BMC Geriatr..

[8] Dadras O., SeyedAlinaghi S., Karimi A., Shamsabadi A., Qaderi K., Ramezani M., Mirghaderi S.P., Mahdiabadi S., Vahedi F., Saeidi S. (2022). COVID-19 mortality and its predictors in the elderly: A systematic review. Health Sci. Rep..

[9] Bull-Otterson L., Baca S., Saydah S., Boehmer T.K., Adjei S., Gray S., Harris A.M. (2022). Post–COVID Conditions Among Adult COVID-19 Survivors Aged 18–64 and ≥65 Years—United States, March 2020–November 2021. Morb. Mortal. Wkly. Rep..

[10] Chen C., Haupert S.R., Zimmermann L., Shi X., Fritsche L.G., Mukherjee B. (2022). Global Prevalence of Post-Coronavirus Disease 2019 (COVID-19) Condition or Long COVID: A Meta-Analysis and Systematic Review. J. Infect. Dis..

[11] WHO (2022). Post COVID-19 Condition (Long COVID). https://www.who.int/europe/news-room/fact-sheets/item/post-covid-19-condition.

[12] Davis H.E., McCorkell L., Vogel J.M., Topol E.J. (2023). Long COVID: Major findings, mechanisms and recommendations. Nat. Rev. Microbiol..

[13] Callard F., Perego E. (2021). How and why patients made Long Covid. Soc. Sci. Med..

[14] Rodriguez-Sanchez I., Rodriguez-Mañas L., Laosa O. (2022). Long COVID-19. Clin. Geriatr. Med..

[15] Nittas V., Gao M., West E.A., Ballouz T., Menges D., Wulf Hanson S., Puhan M.A. (2022). Long COVID Through a Public Health Lens: An Umbrella Review. Public Health Rev..

[16] Domingo F.R., Waddell L.A., Cheung A.M., Cooper C.L., Belcourt V.J., Zuckermann A.M.E., Corrin T., Ahmad R., Boland L., Laprise C. (2021). Prevalence of long-term effects in individuals diagnosed with COVID-19: An updated living systematic review. Epidemiology.

[17] Michelen M., Manoharan L., Elkheir N., Cheng V., Dagens A., Hastie C., O’Hara M., Suett J., Dahmash D., Bugaeva P. (2021). Characterising long COVID: A living systematic review. BMJ Glob. Health.

[18] Nasserie T., Hittle M., Goodman S.N. (2021). Assessment of the Frequency and Variety of Persistent Symptoms Among Patients With COVID-19: A Systematic Review. JAMA Netw. Open.

[19] De Oliveira J.F., De Ávila R.E., De Oliveira N.R., Da Cunha Severino Sampaio N., Botelho M., Gonçalves F.A., Neto C.J.F., Milagres A.C.d.A., Gomes T.C.C., Pereira T.L. (2022). Persistent symptoms, quality of life, and risk factors in long COVID: A cross-sectional study of hospitalized patients in Brazil. Int. J. Infect. Dis..

[20] Salci M.A., Carreira L., Baccon W.C., Marques F.R.D.M., Höring C.F., Oliveira M.L.F.D., Milan N.S., de Souza F.C.S., Gallo A.M., Covre E.R. (2023). Perceived quality of life and associated factors in long COVID syndrome among older Brazilians: A cross-sectional study. J. Clin. Nurs..

[21] Sathyamurthy P., Madhavan S., Pandurangan V. (2021). Prevalence, Pattern and Functional Outcome of Post COVID-19 Syndrome in Older Adults. Cureus.

[22] Daitch V., Yelin D., Awwad M., Guaraldi G., Milić J., Mussini C., Falcone M., Tiseo G., Carrozzi L., Pistelli F. (2022). Characteristics of long-COVID among older adults: A cross-sectional study. Int. J. Infect. Dis..

[23] Tosato M., Carfì A., Martis I., Pais C., Ciciarello F., Rota E., Tritto M., Salerno A., Zazzara M.B., Martone A.M. (2021). Prevalence and predictors of persistence of COVID-19 symptoms in older adults: A single-center study. J. Am. Med. Dir. Assoc..

[24] Heanoy E.Z., Uzer T., Brown N.R. (2022). COVID-19 Pandemic as a Transitional Event: From the Perspective of the Transition Theory. Encyclopedia.

[25] Hossain M.M., Das J., Rahman F., Nesa F., Hossain P., Islam A.M.K., Tasnim S., Faizah F., Mazumder H., Purohit N. (2023). Living with “long COVID”: A systematic review and meta-synthesis of qualitative evidence. PLoS ONE.

[26] Meleis A.I., Sawyer L.M., Im E.O., Hilfinger Messias D.K., Schumacher K. (2000). Experiencing transitions: An emerging middle-range theory. ANS. Adv. Nurs. Sci..

[27] Skilbeck L., Spanton C., Paton M. (2023). Patients’ lived experience and reflections on long COVID: An interpretive phenomenological analysis within an integrated adult primary care psychology NHS service. J. Patient Rep. Outcomes.

[28] Nikolich J.Ž., Rosen C.J. (2023). Toward Comprehensive Care for Long Covid. N. Engl. J. Med..

[29] Zhang Y. (2017). Uncertainty in Illness: Theory Review, Application, and Extension. Oncol. Nurs. Forum.

[30] Dong L., Chen L., Ding S. (2022). Illness uncertainty among patients with COVID-19 in the Mobile Cabin Hospital. Nurs. Open.

[31] Hao F., Tam W., Hu X., Tan W., Jiang L., Jiang X., Zhang L., Zhao X., Zou Y., Hu Y. (2020). A quantitative and qualitative study on the neuropsychiatric sequelae of acutely ill COVID-19 inpatients in isolation facilities. Transl. Psychiatry.

[32] Ladds E., Rushforth A., Wieringa S., Taylor S., Rayner C., Husain L., Greenhalgh T. (2020). Persistent symptoms after Covid-19: Qualitative study of 114 “long Covid” patients and draft quality principles for services. BMC Health Serv. Res..

[33] Chasco E.E., Dukes K., Jones D., Comellas A.P., Hoffman R.M., Garg A. (2022). Brain Fog and Fatigue following COVID-19 Infection: An Exploratory Study of Patient Experiences of Long COVID. Int. J. Environ. Res. Public Health.

[34] Heiberg K.E., Heggestad A.K.T., Jøranson N., Lausund H., Breievne G., Myrstad M., Ranhoff A.H., Walle-Hansen M.M., Bruun-Olsen V. (2022). ‘Brain fog’, guilt, and gratitude: Experiences of symptoms and life changes in older survivors 6 months after hospitalisation for COVID-19. Eur. Geriatr. Med..

[35] Bayeh R., Yampolsky M.A., Ryder A.G. (2021). The Social Lives of Infectious Diseases: Why Culture Matters to COVID-19. Front. Psychol..

[36] United Nations (2015). Transforming Our World: The 2030 Agenda for Sustainable Development. https://sustainabledevelopment.un.org/post2015/transformingourworld.

[37] Salci M.A., Carreira L., Facchini L.A., Oliveira M.L.F., de Oliveira R.R., Ichisato S.M.T., Covre E.R., Pesce G.B., Santos J.A.T., Derhun F.M. (2022). Post-acute COVID and long-COVID among adults and older adults in the State of Paraná, Brazil: Protocol for an ambispective cohort study. BMJ Open.

[38] Charmaz K. (2014). Constructing Ground Theory: A Pratical Guide through Qualitative Analysis.

[39] Charmaz K., Thornberg R. (2021). The pursuit of quality in grounded theory. Qual. Res. Psychol..

[40] Tong A., Sainsbury P., Craig J. (2007). Consolidated criteria for reporting qualitative research (COREQ): A 32-item checklist for interviews and focus groups. Int. J. Qual. Health Care.

[41] Sanchez M., Moura E., Moreira J., Lima R., Barreto I., Pereira C., Santos L. (2021). Mortality from COVID-19 in Brazil: Analysis of death’s civil registry from 2020 January to 2021 February. SciELO Prepr..

[42] WHO (2015). World Report on Ageing and Health. https://iris.who.int/bitstream/handle/10665/186468/WHO_FWC_ALC_15.01_eng.pdf?sequence=1.

[43] Mansell V., Hall Dykgraaf S., Kidd M., Goodyear-Smith F. (2022). Long COVID and older people. Lancet Healthy Longev..

[44] Schiavi M., Fugazzaro S., Bertolini A., Denti M., Mainini C., Accogli M.A., Bedogni G., Ghizzoni D., Esseroukh O., Gualdi C. (2022). “Like before, but not exactly”: The Qualy-REACT qualitative inquiry into the lived experience of long COVID. BMC Public Health.

[45] DeJonckheere M., Vaughn L.M. (2019). Semistructured interviewing in primary care research: A balance of relationship and rigour. Fam. Med. Com. Health.

[46] Carvalho M.R.D.D., Rocha S.S.D., Alvarenga W.A. (2022). (Re)discovering the grounded theory for research in nursing: Reflections on the relativist strand. Rev. Enferm. UERJ.

[47] Rädiker S., Kuckartz U. (2019). Analyse Qualitativer Daten mit MAXQDA.

[48] Jamili S., Ebrahimipour H., Adel A., Badiee Aval S., Hoseini S.J., Vejdani M., Ebnehoseini Z. (2022). Experience of patients hospitalized with COVID-19: A qualitative study of a pandemic disease in Iran. Health Expect..

[49] Bogusz R., Nowakowska L., Majchrowska A., Patryn R., Pawlikowski J., Zagaja A., Kiciński P., Pacyna M., Puacz E. (2022). Convalescents’ Reports on COVID-19 Experience—A Qualitative Study. Int. J. Environ. Res. Public Health..

[50] Sahoo S., Mehra A., Suri V., Malhotra P., Yaddanapudi L.N., Dutt Puri G., Grover S. (2020). Lived experiences of the corona survivors (patients admitted in COVID wards): A narrative real-life documented summaries of internalized guilt, shame, stigma, anger. Asian J. Psychiatry.

[51] Marcinowicz L., Fejfer-Wirbal E., Taranta E., Chlabicz S., Terlikowski S.J. (2022). Experiences and Expectations of Patients Hospitalized for COVID-19: A Qualitative Study in Poland. Int. J. Environ. Res. Public Health.

[52] Garfin D.R., Silver R.C., Holman E.A. (2020). The novel coronavirus (COVID-2019) outbreak: Amplification of public health consequences by media exposure. Health Psychol..

[53] Ahmad A.R., Murad H.R. (2020). The Impact of Social Media on Panic During the COVID-19 Pandemic in Iraqi Kurdistan: Online Questionnaire Study. J. Med. Internet Res..

[54] Venturas M., Prats J., Querol E., Zabalegui A., Fabrellas N., Rivera P., Casafont C., Cuzco C., Frías C.E., Olivé M.C. (2021). Lived Experiences of Hospitalized COVID-19 Patients: A Qualitative Study. Int. J. Environ. Res. Public Health.

[55] Wittenberg E., Goldsmith J.V., Chen C., Prince-Paul M., Johnson R.R. (2021). Opportunities to improve COVID-19 provider communication resources: A systematic review. Patient Educ. Couns..

[56] Hwang T.-J., Rabheru K., Peisah C., Reichman W., Ikeda M. (2020). Loneliness and social isolation during the COVID-19 pandemic. Int. Psychogeriatr..

[57] Haltom T.M., Tiong J., Evans T.L., Kamdar N., True G., Kunik M.E. (2023). Unmet Needs and Coping Strategies of Older Underserved Veterans During the COVID-19 Pandemic. J. Prim. Care Community Health.

[58] Delgado M.C.M. (2022). COVID-19: A family’s perspective. COVID-19 Pandemic.

[59] Gayatri M., Puspitasari M.D. (2022). The Impact of COVID-19 Pandemic on Family Well-Being: A Literature Review. Fam. J..

[60] Da Costa ESilva G.R., Moura W.É.A., Dos Santos K.C., Gomes D.O., Bandeira G.N., Guimarães R.A., Rosso C.F.W., Bazilio G.S., Leite V.R.M.C., Caetano K.A.A. (2023). Long-Term Symptoms after Mild Coronavirus Disease in Healthy Healthcare Professionals: A 12-Month Prospective Cohort Study. Int. J. Environ. Res. Public Health.

[61] Wang Y., Bao S., Chen Y. (2022). The Illness Experience of Long COVID Patients: A Qualitative Study Based on the Online Q&A Community Zhihu. Int. J. Environ. Res. Public Health.

[62] Poyraz B.Ç., Poyraz C.A., Olgun Y., Gürel Ö., Alkan S., Özdemir Y.E., Balkan I.I., Karaali R. (2021). Psychiatric morbidity and protracted symptoms after COVID-19. Psychiatry Res..

[63] Trevisan C., Noale M., Prinelli F., Maggi S., Sojic A., Di Bari M., Molinaro S., Bastiani L., Giacomelli A., Galli M. (2021). Age-Related Changes in Clinical Presentation of Covid-19: The EPICOVID19 Web-Based Survey. Eur. J. Intern. Med..

[64] Mullard J.C.R., Kawalek J., Parkin A., Rayner C., Mir G., Sivan M., Greenhalgh T. (2023). Towards evidence-based and inclusive models of peer support for long covid: A hermeneutic systematic review. Soc. Sci. Med..

[65] Sun W., Chen W.-T., Zhang Q., Ma S., Huang F., Zhang L., Lu H. (2021). Post-Traumatic Growth Experiences among COVID-19 Confirmed Cases in China: A Qualitative Study. Clin. Nurs. Res..

[66] Jiang D.H., Roy D.J., Gu B.J., Hassett L.C., McCoy R.G. (2021). Postacute Sequelae of Severe Acute Respiratory Syndrome Coronavirus 2 Infection. JACC Basic Transl. Sci..

[67] Kohn L., Dauvrin M., Detollenaere J., Primus-de Jong C., Maertens De Noordhout C., Castanares-Zapatero D., Cleemput I., Heede K.V.D. (2022). Long COVID and return to work: A qualitative study. Occup. Med..

[68] Laranjeira C., Querido A. (2022). Hope and Optimism as an Opportunity to Improve the “Positive Mental Health” Demand. Front. Psychol..

[69] Collazo-Castiñeira P., Rodríguez-Rey R., Garrido-Hernansaiz H., Collado S. (2022). Prediction of post-traumatic growth in the face of the COVID-19 crisis based on resilience, post-traumatic stress and social participation: A longitudinal study. Front. Psychol..

[70] Gibbs R.W. (2020). How metaphors shape the particularities of illness and healing experiences. Transcult. Psychiatry.

[71] Lindmark U., Bülow P.H., Mårtensson J., Rönning H., A.D.U.L.T. Research Group (2019). The use of the concept of transition in different disciplines within health and social welfare: An integrative literature review. Nurs. Open..

[72] Vyas L. (2022). “New normal” at work in a post-COVID world: Work–life balance and labor markets. Policy Soc..

[73] Shirmohammadi M., Au W.C., Beigi M. (2022). Remote work and work-life balance: Lessons learned from the covid-19 pandemic and suggestions for HRD practitioners. Hum. Resour. Dev. Int..

[74] Martínez M., Luis E.O., Ceric F., Bermejo-Martins E. (2023). Editorial: Mental health promotion during COVID-19: Applications from self-care resources, lifestyles, and environments. Front. Psychol..

[75] Sánchez-Hernández Ó., Barkavi-Shani M., Bermejo R.M. (2022). Promotion of Resilience and Emotional Self-Care in Families and Health Professionals in Times of COVID-19. Front. Psychol..

[76] Fang C., Baz S.A., Sheard L., Carpentieri J. (2023). ‘I am just a shadow of who I used to be’—Exploring existential loss of identity among people living with chronic conditions of Long COVID. Sociol. Health Illn..

[77] Laranjeira C., Querido A. (2023). An in-depth introduction to arts-based spiritual healthcare: Creatively seeking and expressing purpose and meaning. Front. Psychol..

[78] Walsh F. (2020). Loss and Resilience in the Time of COVID-19: Meaning Making, Hope, and Transcendence. Fam. Proc..

[79] Sen H.E., Colucci L., Browne D.T. (2022). Keeping the Faith: Religion, Positive Coping, and Mental Health of Caregivers During COVID-19. Front. Psychol..

[80] Thomas C., Faghy M.A., Owen R., Yates J., Ferraro F., Bewick T., Haggan K., Ashton R.E.M. (2023). Lived experience of patients with Long COVID: A qualitative study in the UK. BMJ Open.

[81] Gorna R., MacDermott N., Rayner C., O’Hara M., Evans S., Agyen L., Nutland W., Rogers N., Hastie C. (2021). Long COVID guidelines need to reflect lived experience. Lancet.

